# Nog steeds ‘gewoon’ wetenschap

**DOI:** 10.1007/s13629-021-9003-9

**Published:** 2021-10-23

**Authors:** Peter F.A. Mulders

**Affiliations:** Radboudumc, Nijmegen, Nederland

## Abstract

Voor u ligt weer het volgende nummer van Tijdschrift voor Urologie. Dit keer met de abstracts van de Najaarsvergadering van de NVU. We zijn er inmiddels aan gewend dat we elkaar minder vaak zien, dat de interactie nog steeds veelal digitaal is en dat er nog steeds wetenschap wordt bedreven. Interessant is het om te zien of de inhoud van de abstracts anders is geworden. De urologische praktijk wél. Hoe? Dat weten we nog niet in detail. Andere prioriteiten, andere volgorde?

Voor u ligt weer het volgende nummer van Tijdschrift voor Urologie. Dit keer met de abstracts van de Najaarsvergadering van de NVU. We zijn er inmiddels aan gewend dat we elkaar minder vaak zien, dat de interactie nog steeds veelal digitaal is en dat er nog steeds wetenschap wordt bedreven. Interessant is het om te zien of de inhoud van de abstracts anders is geworden. De urologische praktijk wél. Hoe? Dat weten we nog niet in detail. Andere prioriteiten, andere volgorde?

De arts-assistenten, arts-onderzoekers en urologen hebben weer zeer divers onderzoek opgestuurd. De wetenschappelijke commissie heeft zich weer van zijn taak gekweten en het meest relevante onderzoek geaccepteerd voor de vergadering, hoe hybride die dan ook wordt gehouden. Nog steeds is prostaatkanker ons nummer één interessegebied met zijn aparte sessie. Ook hierbinnen zijn er verschillende interessegebieden, maar die zijn nog wel vaak gerelateerd aan de PSMA/PET. Hopelijk zal er tijdens de jaarvergadering discussie ontstaan over hoe we die scan in de dagelijkse praktijk gaan implementeren.

Weinig echter wordt COVID-19 binnen de urologie onderzocht. Geen enkele urologische aandoening is aan deze ziekte gerelateerd. Was hematurie bijvoorbeeld een gevolg van COVID-19, dan had het bestuur of de commissie externe betrekkingen regelmatig aan de vele talkshowtafels gezeten, en hadden we vast meer BN’ers onder ons. Maar nu moeten we het doen met de onderlinge gesprekken tijdens de najaarsvergadering.

Met dit nummer kunt u zich op die vergadering voorbereiden en de presentaties van kritische vragen en nuttige suggesties voorzien. Met elkaar kunnen we de abstracts laten uitgroeien tot een waar artikel, wellicht ook weer ter publicatie in dit tijdschrift. We zijn benieuwd, maar we zijn vooral benieuwd hóe we elkaar weer gaan zien. Terug naar het normaal? Laten we ervan uitgaan dat de ervaring van digitaal werken zich blijft vertalen naar meer mogelijkheden voor het vergaren van kennis die niet eindigt in een fileborrel. Wie weet.

Nogmaals heel veel leesplezier en ‘tot corona’, tot computerscherm of fysiek in welke vorm dan ook.

**Peter F.A. Mulders –** uroloog

